# Atrial fibrillation in end‐stage heart failure: Cellular mechanisms behind CASTLE‐HTx

**DOI:** 10.1002/ejhf.70051

**Published:** 2025-09-17

**Authors:** Maria Knierim, Nico Hartmann, Wiebke Maurer, Steffen Pabel, Simon Sedej, Dirk von Lewinski, Jan Gummert, Christian Sohns, Katrin Streckfuss‐Bömeke, Samuel Sossalla

**Affiliations:** ^1^ Clinic for Cardio‐Thoracic and Vascular Surgery University Medical Center Göttingen Göttingen Germany; ^2^ Clinic for Cardiology and Pneumology, University Medical Center Göttingen, and DZHK (German Centre for Cardiovascular Research), partner site Göttingen Göttingen Germany; ^3^ Institute of Cardiovascular Physiology University Medical Center Göttingen Göttingen Germany; ^4^ Center for Systems Biology Massachusetts General Hospital and Harvard Medical School Boston MA USA; ^5^ Department of Cardiology Medical University of Graz Graz Austria; ^6^ BioTechMed‐Graz Graz Austria; ^7^ Faculty of Medicine University of Maribor Maribor Slovenia; ^8^ Clinic for Thoracic and Cardiovascular Surgery, Herz‐ und Diabeteszentrum NRW, Ruhr‐University Bochum Bad Oeynhausen Germany; ^9^ Clinic for Electrophysiology, Herz‐ und Diabeteszentrum NRW, Ruhr‐University Bochum Bad Oeynhausen Germany; ^10^ Institute of Pharmacology and Toxicology Julius‐Maximilians‐University Würzburg Germany; ^11^ Medical Clinic I, Cardiology and Angiology Justus‐Liebig‐University Gießen Germany; ^12^ Department of Cardiology at Kerckhoff Heart and Lung Center Bad Nauheim, Germany and DZHK (German Center for Cardiovascular Research), Partner Site RheinMain Frankfurt Germany

Atrial fibrillation (AF) often coexists in patients with heart failure (HF). Rhythm restoration is increasingly recognized as a prognostically relevant strategy in patients with HF and AF. However, compared to other frequent comorbidities like chronic kidney disease or type 2 diabetes, the reciprocal interaction between HF and AF and particularly the impact of AF on the development and progression of HF has not been fully understood and continues to be underestimated.^1^

Recently, the CASTLE‐HTx trial, among others, demonstrated that catheter ablation for AF and the reduction of AF burden improved prognosis even in patients with end‐stage HF. This was associated with improved left ventricular systolic function following AF ablation.^1^, ^2^, ^3^ Those results clearly underscore the need to explore the largely unknown mechanisms of AF in end‐stage HF.

To investigate the effects of persisting AF on ventricular function in severe HF, we isolated 15 human ventricular trabeculae from freshly explanted end‐stage HF hearts from nine patients in total and seven trabeculae from four non‐HF organ donors (non‐failing [NF]) as described previously.^4^ NF samples were acquired from patients whose hearts could not be transplanted due to non‐cardiac medical or technical reasons. The cohort of HF patients had a mean age of 60.4 ± 1.6 years (mean ± standard error of the mean), left ventricular ejection fraction of 28.0 ± 3.2% and ischaemic cardiomyopathy in 44.4% of cases. All patients received guideline‐directed medical therapy with no significant differences. The isolated trabeculae were subjected to AF simulation by electric stimulation *in vitro* for up to 8 h using a purpose‐made pacing system (C‐Pace EM, IonOptix, Westwood, MA, USA) with irregular stimulation intervals (60 bpm with 40% beat‐to‐beat variability) versus control stimulation (60 bpm with regular intervals). Contractility was assessed at 60 bpm in each group at baseline and intermittently after 2, 4, and 8 h of continuous pacing.

To investigate cellular mechanisms of AF in human HF, we utilized a total of nine differentiations of human induced pluripotent stem cell‐derived cardiomyocytes (hiPSC‐CM) generated from two patients with familial dilated cardiomyopathy (DCM) with end‐stage HF and three differentiations from one healthy control patient.^5^ Cardiomyocyte (CM) differentiation and cell culture were performed as previously described.^5^, ^6^ Chronic AF simulation for 48 h was performed by continuous pacing of hiPSC‐CM in culture using the C‐Pace EM pacing system (90 bpm with 30% beat‐to‐beat variability vs. regular 60 bpm).

Intracellular Ca^2+^ cycling was studied in DCM and healthy hiPSC‐CM after 48 h of AF simulation using the Ca^2+^ sensitive dye Fura‐2 as described elsewhere.^6^

The patch‐clamp method in voltage‐clamp mode was employed to record action potentials (AP) in DCM and healthy hiPSC‐CM after 48 h of AF or control stimulation, following an established protocol.^6^

All procedures were performed according to the Declaration of Helsinki and were approved by the local ethics committees of the University of Göttingen (ref. no. 10/9/15, 31/9/00) and Medical University of Graz (ref. no. 28‐508 ex 15/16).

**Figure 1:**
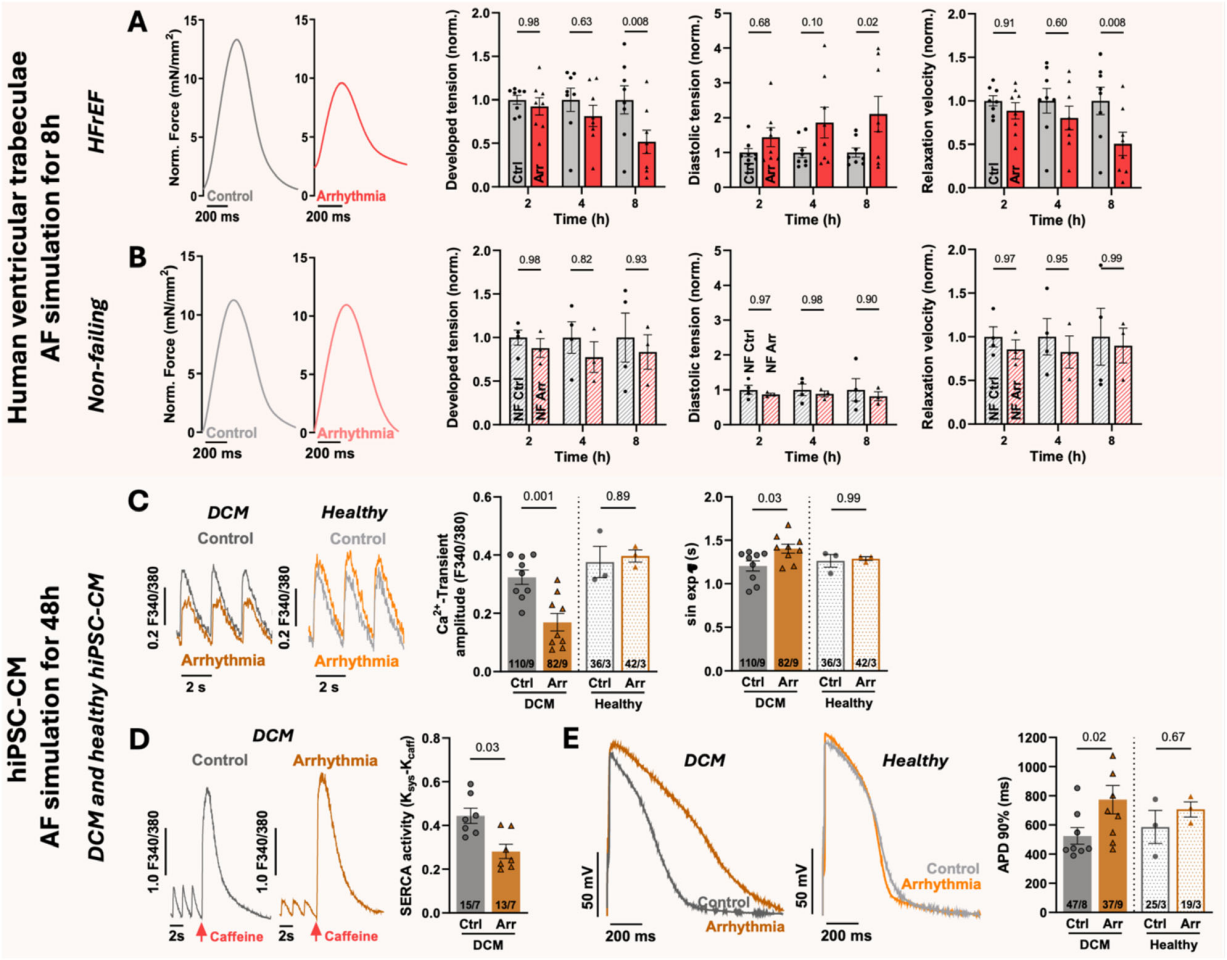
Effects of atrial fibrillation (AF) on human ventricular myocardium in end‐stage heart failure. (*A*) Original twitch recordings after 8 h of AF simulation or control stimulation, mean values of normalized developed force, diastolic tension and relaxation velocity in human ventricular trabeculae from end‐stage heart failure patients after 2, 4, and 8 h of AF simulation (arrhythmia [Arr], 60 bpm with 40% beat‐to‐beat variability) or control stimulation (control [Ctrl], 60 bpm). *N* = 8 trabeculae/7 patients control stimulation versus 8/8 AF simulation. (B) Respective experiments and original twitches, mean values of normalized developed force, diastolic tension and relaxation velocity in ventricular trabeculae from non‐heart failure organ donors. *N* = 4 trabeculae/3 donors control stimulation versus 3/3 AF simulation. (*C*) Chronic AF simulation (Arr, 90 bpm with 30% beat‐to‐beat variability) or control stimulation (60 bpm) in human‐induced pluripotent stem cell‐derived cardiomyocytes (hiPSC‐CM) from two familial dilated cardiomyopathy (DCM) patients and one healthy donor. Original Ca^2+^ transients, mean values of Ca^2+^ transient amplitude and Ca^2+^ transient decay time (sin exp τ). *N* = 110 cardiomyocytes/9 differentiations DCM control stimulation versus 82/9 DCM AF simulation and 36/3 healthy control stimulation versus 42/3 healthy AF simulation. (*D*) Caffeine‐induced Ca^2+^ transients and mean values of SERCA activity (K_sys_–K_caff_) in DCM hiPSC‐CM after AF simulation. *N* = 15 cardiomyocytes/7 differentiations DCM control stimulation versus 13/7 DCM AF simulation. (*E*) Patch‐clamp action potential recordings and mean values of action potential duration (APD) 90% in DCM and healthy hiPSC‐CM after chronic AF simulation. *N* = 47 cardiomyocytes/8 differentiations DCM control stimulation versus 37/9 DCM AF simulation and 25/3 healthy control stimulation versus 19/3 healthy AF simulation. Statistical testing was performed as follows: *A* + *B*: two‐way ANOVA with Sidak's correction for multiple comparisons; *D*: nested *t*‐test; *C* + *E*: nested one‐way ANOVA with Sidak's correction for multiple comparisons. *P*‐values are indicated in each figure and considered statistically significant if *p* < 0.05. HFrEF, heart failure with reduced ejection fraction; NF, non‐failing.

Atrial fibrillation simulation for 8 h resulted in a substantial decrease in systolic contractile force in human end‐stage HF trabeculae compared to control (*Figure* *1A*). Furthermore, diastolic function rapidly deteriorated due to AF simulation, as represented by a significant increase in diastolic tension and slowed relaxation compared to control stimulation (*Figure* *1A*). In contrast, trabeculae from NF hearts did not exhibit a negative contractile response to AF simulation (*Figure* *1B*), demonstrating the rapid deterioration of ventricular function solely because of AF in pre‐existing HF.

Ca^2+^ cycling measurements were performed in DCM and healthy hiPSC‐CM after 48 h of AF simulation or rhythmic control stimulation to investigate potential underlying cellular mechanisms. Continuous AF simulation for 48 h in DCM hiPSC‐CM resulted in a substantial reduction of systolic Ca^2+^ release compared to control stimulation, as measured by reduced Ca^2+^ transient amplitude (*Figure* *1C*). Concomitantly, the intracellular Ca^2+^ elimination in diastole (elimination constant τ) was significantly slower after AF simulation than upon control stimulation in DCM hiPSC‐CM (*Figure* *1C*). The latter was likely related to a reduction in SERCA2a activity after AF simulation, leading to slower Ca^2+^ reuptake to the sarcoplasmic reticulum as evaluated by rapid high‐dose caffeine application (*Figure* *1D*). Again, these effects could not be observed in healthy hiPSC‐CM after 48 h of AF simulation (*Figure* *1C*).

Furthermore, patch‐clamp experiments in DCM hiPSC‐CM revealed a significant prolongation of the AP duration (90%) after 48 h of AF simulation compared to rhythmic stimulation in DCM hiPSC‐CM, but not in healthy hiPSC‐CM (*Figure* *1E*). These effects altogether illustrate the detrimental impact of AF on human failing myocardium compared to healthy myocardium.

Our translational human myocardium‐based experiments demonstrate that AF leads to rapid and severe ventricular contractile dysfunction and cellular functional remodelling in human HF. As Ca^2+^ is a major determinant of myocardial contractile function, this could be explained mechanistically by adverse Ca^2+^ cycling alterations in DCM hiPSC‐CM in early stages of AF simulation. Furthermore, AF simulation induced a distinct prolongation of the AP in hiPSC‐CM, which constitutes an electrophysiological hallmark of HF. Interestingly, in NF myocardium and healthy hiPSC‐CM, these effects could not be observed.

Recent experimental work from our group has shown similar HF‐typical effects of long‐term AF simulation on Ca^2+^ handling and AP morphology, although only after a longer period of AF simulation of 7 days in healthy CM.^6^, ^7^ Mechanistically, increased production of reactive oxygen species with oxidative regulation of Ca^2+^/calmodulin‐dependent protein kinase II (CaMKII), along with differential regulation of proteins involved in Ca^2+^ homeostasis (e.g. reduction of SERCA2a activity) and electrophysiology, have been demonstrated as causal mechanisms for the observed alterations.^6^ Supporting this pathway and the importance of AF‐induced alterations, increased CaMKII and AMP‐activated protein kinase activity after AF simulation have been associated with Ca^2+^ cycling alterations and disturbed fatty acid and glucose metabolism, accompanied by pro‐apoptotic pathways in a neonatal rat‐CM model.^8^

Most noteworthy, we here demonstrate that in pre‐existing HF adverse effects of AF on myocardial function can be observed earlier and to a greater extent than in healthy myocardium. This highlights the vulnerability of HF ventricular myocardium towards AF and, in context with recent clinical studies like CASTLE‐AF or CASTLE‐HTx, underscores the importance of timely rhythm restoration and AF burden reduction in pre‐existing HF.
